# Successful Perimortem Cesarean Section With Cardiopulmonary Resuscitation and Anesthesia: A Report of an Unique Case

**DOI:** 10.7759/cureus.55037

**Published:** 2024-02-27

**Authors:** Tomoyuki Iwai, Shinichi Inomata

**Affiliations:** 1 Department of Anesthesiology, University of Tsukuba Hospital, Tsukuba, JPN; 2 Department of Anesthesiology, Division of Clinical Medicine, Institute of Medicine, University of Tsukuba, Tsukuba, JPN

**Keywords:** severe pulmonary edema, emergent perimortem cesarean delivery, pregnancy-induced hypertension (pih), cpr in pregnancy, intrapartum maternal cardiac arrest

## Abstract

Cardiac arrest after acute pulmonary edema in pregnancy is an uncommon event but one with a potentially disastrous outcome. We report the case of a pregnant woman with preeclampsia who presented with rapidly advancing pulmonary edema and subsequently went into cardiac arrest on arrival at the operating room. A perimortem cesarean section was performed in addition to cardiopulmonary resuscitation and anesthesia. These simultaneous treatments resulted in excellent maternal and neonatal outcomes. Moreover, therapeutic brain hypothermia was performed. To our knowledge, this is the first case report of a patient undergoing a cesarean section during cardiac arrest and treated with brain hypothermia. We discuss some of the issues arising from the case in this report.

## Introduction

Cardiopulmonary arrest in pregnant women is an uncommon event, with an estimated incidence of one in 30,000 pregnancies [1]. However, once a pregnant woman goes into cardiopulmonary arrest, the prognosis is worse than in other populations, with a reported survival rate of 6.9% [1,2]. Treatment of a collapsed pregnant woman differs in several respects from normal resuscitation of an adult patient. These differences include early securing of the airway by tracheal intubation, release of the inferior vena cava compression caused by an enlarged uterus, and making a decision regarding perimortem cesarean delivery (within the first four minutes) [2,3].

Pregnancy-induced hypertension (PIH) is a possible complication of pregnancy. The incidence rate of PIH was reported to be 6.3% [4]. In Japan, PIH is classified into gestational hypertension, preeclampsia, or eclampsia. Other countries have slight variations in this classification. Treatments for PIH include bed rest, dietary measures, medication (antihypertensives, anticonvulsants, magnesium, anticoagulants), and eventually termination of pregnancy.

Here, we report a case of cardiac arrest on arrival at the operating room caused by severe preeclampsia followed by rapidly worsening pulmonary edema. Fortunately, we secured successful outcomes for both the mother and the neonate.

## Case presentation

**Figure 1:**
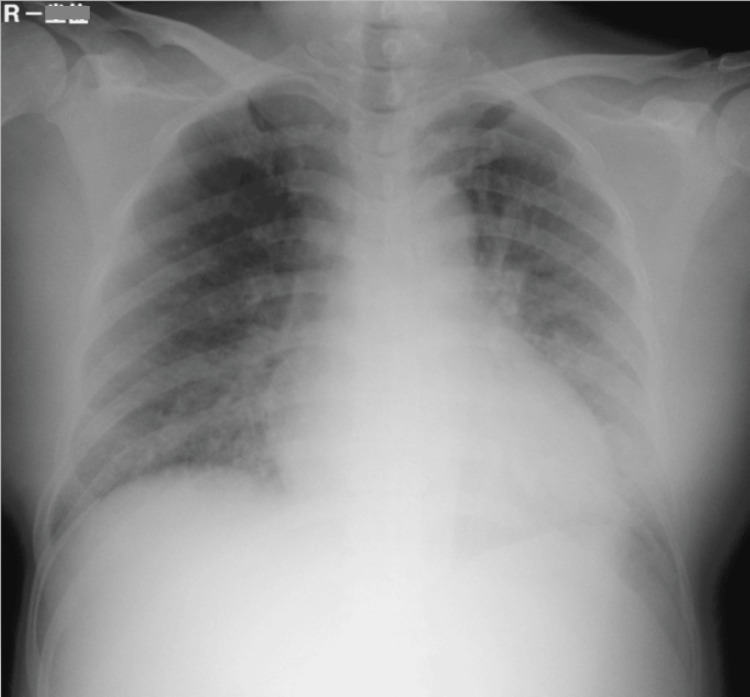
Chest X-ray shows bilateral opacities and butterfly shadow

A 37-year-old pregnant woman, gravida 1, para 1, was admitted to our obstetrics department at 32 weeks gestation. She had delivered a baby by normal vaginal birth when she was 28 years of age. For this pregnancy, she had been started on 750 mg per day of oral methyldopa at 31 weeks because PIH had been identified. One week later, the hypertension had not improved, and her attending doctor decided to admit her to the hospital. The dose of oral methyldopa was increased to 1500 mg per day, and 20 mg per day of oral nifedipine was added. During morning rounds on the seventh day after admission, her physical status was normal. However, at 11:20 AM, her blood pressure was 164/100 mmHg, and she complained of difficulty in breathing. Her oxygen saturation was 89% on room air; therefore, 4-L/min oxygen was applied by mask, which improved the oxygen saturation to 95%. Chest-X ray (Figure 1) and blood gas analysis were performed.

**Figure 2:**
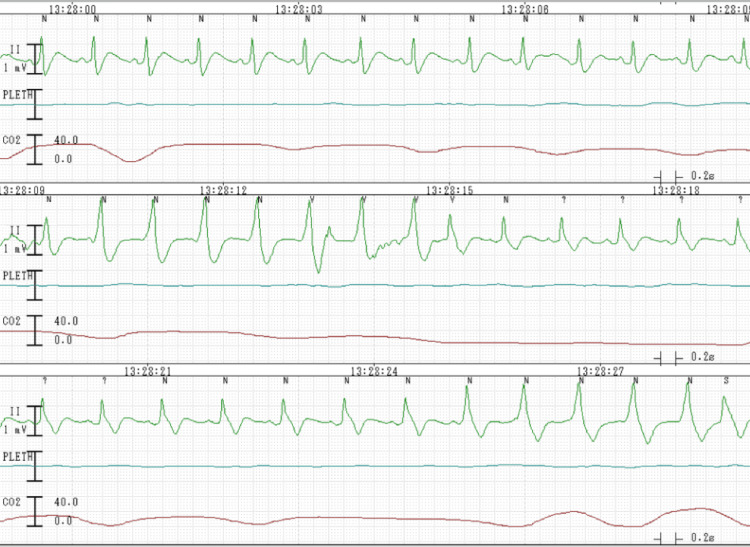
ECG shows nonsustained ventricular tachycardia

Pulmonary edema due to severe preeclampsia was diagnosed, and the obstetrician decided on an immediate cesarean delivery. The obstetrician called the operating department to prepare for urgent cesarean delivery. However, he saw no need for general anesthesia and considered that he had enough time to wait before performing regional anesthesia, which is preferred for most cesarean deliveries because the baby is exposed to the lowest amount of medication. Immediately after he called the operating team, an anesthesia resident visited the maternity ward to perform a preoperative evaluation and to obtain informed consent. When he arrived, the patient’s respiratory condition had deteriorated drastically: she was orthopneic and her oxygen saturation had deteriorated to 60% on 10-L/miuten oxygen by mask. The anesthesia resident decided to transfer the patient to the emergency cesarean section room immediately, and a staff anesthesiologist instructed the nurses to make rapid preparations for immediate surgery. On the patient’s arrival at the operating room, the heartbeat was indistinct at 170 beats/minute and the pulse was difficult to detect. Her ECG showed nonsustained ventricular tachycardia (Figure 2).

**Figure 3:**
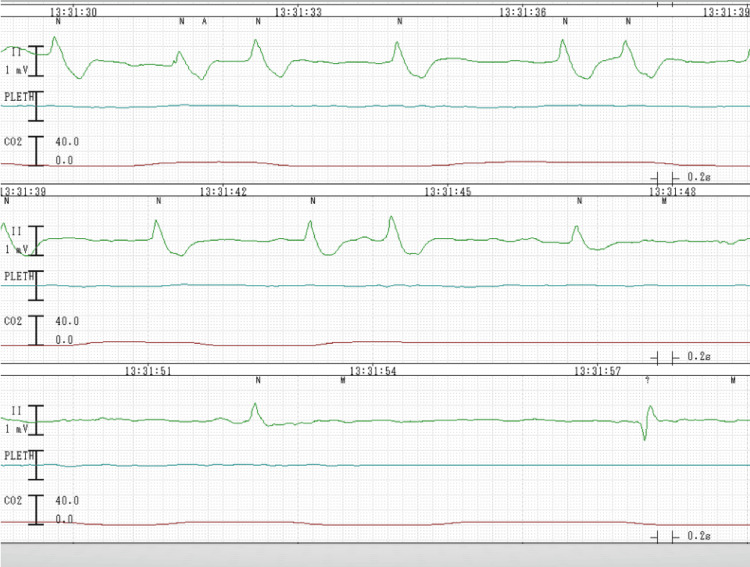
ECG shows slow idioventricular rhythm

On the spot, the staff anesthesiologist started chest compression, and general anesthesia was induced in rapid sequence fashion with thiamylal and rocuronium, followed by tracheal intubation, allowing cesarean section to be performed four minutes after arrival. Her airway was filled with pink bubbles, and she required high positive end-expiratory pressure (PEEP) of 20 cmH2O to suppress the bubbles. Forty seconds after the incision, the neonate was delivered; simultaneously, the patient became pulseless with a slow idioventricular rhythm on ECG (Figure 3).

**Figure 4:**
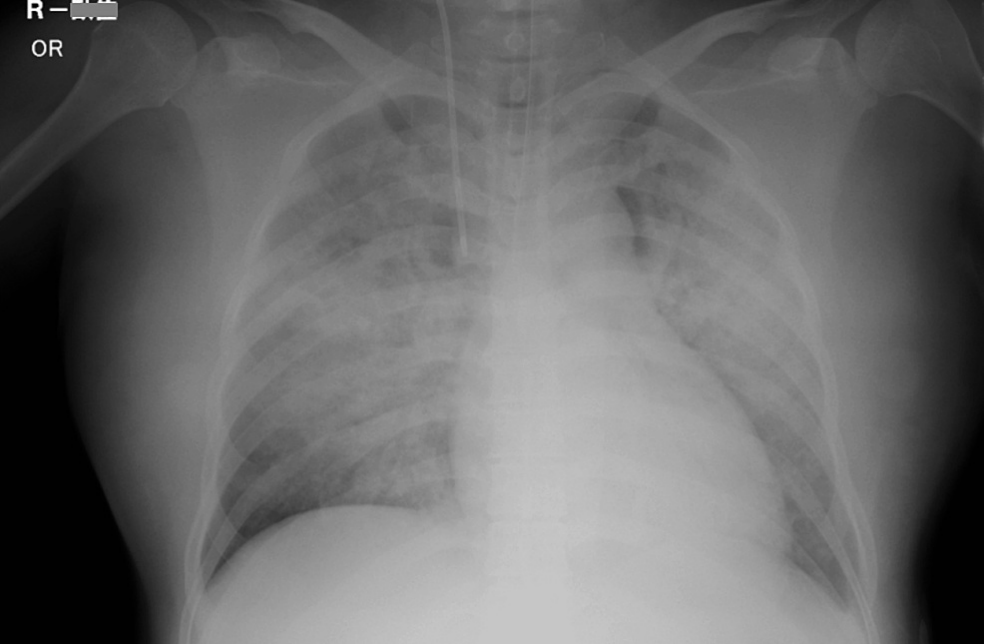
Postoperative chest X-ray shows massive pulmonary edema

Cardiopulmonary resuscitation (CPR) was continued following an advanced cardiovascular life support (ACLS) algorithm. After five minutes of CPR, she received 2 mg of epinephrine, and her ECG pattern showed ventricular fibrillation. She needed defibrillation twice and her ECG pattern returned to a sinus rhythm. Return of spontaneous circulation was achieved 10 minutes after delivery. Wound closure and placement of a central line and an arterial line were performed simultaneously. After the operation, the patient was transferred to the intensive care unit, where she required inotropic support and high PEEP ventilation with a high concentration of oxygen. Figure 4 shows a chest X-ray done after the emergency surgery.

The patient received hypothermic cerebroprotection for four days and was extubated 10 days after the initial event. She was discharged from our hospital 66 days after the event and transferred to a rehabilitation hospital. Seven months later, her only issue was a deficit of short-term memory, with no restrictions on her activities of daily living. The neonate was born with Apgar scores of 3 and 6 at 1 and 5 minutes, respectively, and was intubated on the spot. However, he was discharged from our hospital 29 days after delivery with no clinical evidence of neurologic injury.

## Discussion

Our case offers several important learning points. The etiologies of cardiopulmonary arrest in pregnant women include, in addition to general causes, those specific to the gravid state, ie, preeclampsia/eclampsia, iatrogenic hypermagnesemia, pulmonary embolism, amniotic embolism, and trauma/hemorrhage [5].

In our case, cardiac arrest resulted from severe hypoxemia secondary to deteriorative pulmonary edema. Pulmonary edema is known as a rare but serious complication of eclampsia [6,7]. There was an earlier case report of a pregnant woman who suffered pulmonary edema and fell into cardiopulmonary arrest [8]. She underwent cardiopulmonary resuscitation and perimortem cesarean section. However, it had taken one night from arrival at the hospital for her to collapse. In our case, hypoxemia progressed at an unexpected speed. The patient’s status was normal during morning rounds; nevertheless, she fell into cardiac arrest just two hours after the onset of her subjective symptom of dyspnea. Her circulatory status collapsed very rapidly. Therefore, in the event of a consultation or call about an emergency cesarean section relating to pulmonary edema secondary to eclampsia, anesthesiologists must make a great effort to enable the patient to undergo surgery as soon as possible.

This event occurred on a weekend, and the regular staff of the operating department were on holiday shifts. Only two anesthesiologists and three nurses were stationed in the operating department. We always reserve an emergency cesarean section room that is available 24 hours a day. The room and infant incubator are warmed, and all equipment is ready for use. Anesthetic drugs can be used immediately. This kind of arrangement can be very useful in such an extreme emergency situation.

To our knowledge, the use of brain therapeutic hypothermia for a pregnant woman who has undergone cardiac arrest and cesarean section has not been evaluated in detail. Consensus is still lacking on this topic and in such a situation and therefore, clinicians should practice according to the most recent general recommendations. 

In this case, the patient required high PEEP ventilation because of massive pulmonary edema. After tracheal intubation, the PEEP suppressed the bubbles and preserved oxygenation. Had there been time and resources before she fell into cardiac arrest, noninvasive positive pressure ventilation could have been attempted. This in turn may have allowed us to gain time or improve the progress [9,10].

## Conclusions

We presented a case of cardiac arrest of a pregnant woman during an emergency cesarean section that had favorable maternal and neonatal outcomes. The immediate cause of cardiac arrest was massive pulmonary edema secondary to uncontrolled pregnancy-induced hypertension. The symptoms progressed with remarkable speed, and the patient arrived at the operating room just before the cardiac arrest. This good outcome was due to the immediate transfer of the patient to the operating room and the hospital’s arrangement of maintaining a room for emergency cesarean section.

All hospitals that care for obstetric patients should keep an operating room readily available for emergency cesarean section. To save mothers and children in an extremely hurried situation, such hospitals will require training and cooperation between anesthesiology, obstetrics, operating room, and neonatology personnel.
